# Supersaturated Liquid Formulation of Pazopanib Hydrochloride Loaded with Synergistic Precipitation Inhibitors

**DOI:** 10.3390/molecules29225267

**Published:** 2024-11-07

**Authors:** Jin Woo Park, Sa-Won Lee, Jun Hak Lee, Jun-Pil Jee, Han-Joo Maeng, Dong-Jin Jang, Kwan Hyung Cho

**Affiliations:** 1 College of Pharmacy and Inje Institute of Pharmaceutical Sciences and Research, Inje University, 197 Inje-ro, Gimhae 50834, Republic of Korea; wlsdn4361@naver.com (J.W.P.); dlwnsgkr2341@gmail.com (J.H.L.); 2 Department of Pharmaceutical Engineering, Inje University, 197 Inje-ro, Gimhae 50834, Republic of Korea; lsw314@inje.ac.kr; 3 College of Pharmacy, Chosun University, 309 Pilmun-daero, Dong-gu, Gwangju 61452, Republic of Korea; jee@chosun.ac.kr; 4 College of Pharmacy, Gachon University, Incheon 21936, Republic of Korea; hjmaeng@gachon.ac.kr; 5 Department of Bio-Health Technology, College of Biomedical Science, Kangwon National University, Chuncheon 24341, Republic of Korea

**Keywords:** pazopanib hydrochloride, supersaturated formulation, precipitation inhibitor, dissolution, solubility

## Abstract

This study aimed to develop a supersaturated liquid formulation (SSLF) to enhance the solubility and dissolution of pazopanib hydrochloride (PZH). SSLFs were prepared by a simple stirring method in a heated silicon oil bath (70 °C). PZH showed highly pH-dependent solubility (pH 1.2 > water >> pH 4.0 and pH 6.8) at 37 °C. The SSLF containing glycerol and polyvinylpyrrolidone K30 (PVP K30) increased PZH dispersion solubility (273.66 ± 48.91 μg/mL) at pH 6.8 by more than 50-fold compared with that of glycerol alone (<5 μg/mL), and the PZH precipitate particle size was considerably small (<100 nm). Moreover, the dispersion solubility of PZH from SSLF containing additional propylene glycol (PG) increased to 364.41 ± 2.47 μg/mL. The optimized SSLF10 (PZH/glycerol/PG/PVP K30 = 10/50/20/20, *w*/*w*) exhibited a high dissolution rate at pH 4.0 (>90%) and 6.8 (>55%) until 360 min, whereas PZH powder and PZH glycerol solution showed pH-dependent, low dissolution rates (<10%) under similar conditions. The supersaturation ratio of SSLF10 was very high at 29.88 and 18.36 at pH 6.8 and 4.0, respectively, indicating a stable PZH supersaturation solution. In the transmission electron microscopy analysis, PVP K30 and PG in SSLF10 synergistically suppressed PZH precipitation and recrystallization with small amorphous particles (<200 nm). Therefore, SSLF10 would be a promising formulation with enhanced solubility and dissolution rates regardless of medium pH.

## 1. Introduction

Pazopanib hydrochloride (PZH), 5-[[4-[(2,3-dimethylindazol-6-yl)-methylamino]pyrimidin-2-yl]amino]-2-methylbenzenesulfonamide hydrochloride, is a multi-targeted tyrosine kinase inhibitor for vascular endothelial growth factor receptor, platelet-derived growth factor receptor, and stem cell factor receptor (c-kit), exhibiting excellent anti-tumor and anti-angiogenesis activity both in vitro and in vivo [1]. PZH has been clinically used as a preferred first-line targeted therapy for advanced soft tissue sarcoma and renal cell carcinoma, owing not only to its favorable efficacy but also the low incidence of side effects, including mucositis, hand-foot syndrome, fatigue, and hematologic toxicity [2].

**Figure 1:**
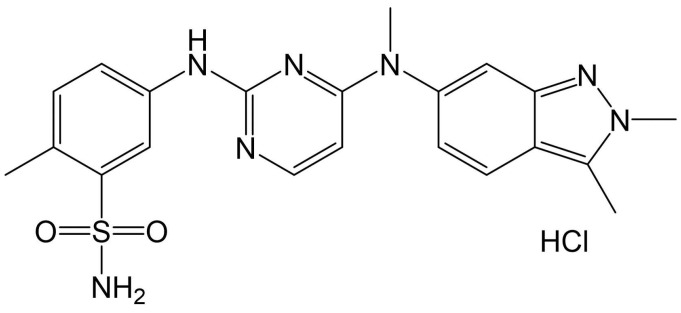
Chemical structure of PZH.

PZH is a Biopharmaceutics Classification System Class II anti-cancer drug that exhibits low oral bioavailability (14–39%) owing to the poor aqueous solubility [1,3]. The dose strength of the commercial product Votrient^®^ (Novartis, Basel, Switzerland) is 200 or 400 mg, with a daily maximum dose reaching up to 800 mg. Drugs with poor oral absorption rates often induce large inter-patient variability in bioavailability, leading to issues of either delayed efficacy or severe side effects due to high plasma concentrations [4]. Particularly, PZH has an alkaline structure similar to that of sulfonamide and pyrimidine (Figure 1), resulting in acute pH-dependent solubility, with insolubility at pH levels higher than 4 [5]. Therefore, PZH molecules dissolved in the acidic conditions of the stomach may precipitate and recrystallize at increased intestinal pH. Moreover, when administered orally, the effect of food causes significant variability in gastrointestinal emptying time and pH; thus, commercial products should be administered 1 h before or 2 h after a meal [6]. Therefore, developing formulations that increase solubility and dissolution rate, regardless of gastrointestinal pH and food intake, is necessary.

Various formulation strategies, such as polymer mixture, lipid-based nanovesicle, and self-nanoemulsifying drug delivery systems (SNEDDSs), have been adopted to increase the solubility and bioavailability of PZH [3,7,8,9]. These technologies have some limitations, including low PZH loading percentage in formulation, insufficient dissolution rate at an intestinal pH of 6.8 without solubilizer aid, and hurdles in mass production and quality control. Thus, developing alternative formulations considering these aspects are still required.

Supersaturated drug delivery systems (SDDSs) are a promising strategy to enhance the solubility and bioavailability of poorly water-soluble drugs. SDDSs incorporate the drug in a rapid-dissolving form, such as solid dispersions and self-nanoemulsifying drug delivery systems (SNEDDSs), resulting in intraluminal concentrations exceeding the apparent solubility of the drug in a saturated state [10]. Supersaturation is underpinned by the “spring” and “parachute” effects. The phenomenon known as the spring effect occurs in thermodynamically unstable high-energy medications, leading to the formation of supersaturated solutions of drugs. In contrast, the parachute effect utilizes polymers or surfactants to maintain the supersaturated state for a desirable duration. Supersaturation is a metastable state that can induce drug precipitation. Therefore, supersaturation stability and the resulting pattern of drug precipitation in the formulation or exposure to an aqueous medium (anti-solvent) are crucial factors in SDDS development. Hence, the important points in the study of SDDSs are physical stability within the formulation, formation of supersaturated concentrations, and precipitation inhibition, which is achievable using a precipitation inhibitor.

Precipitation inhibitors in the preparation of SDDSs suppress drug precipitation in the gastrointestinal tract, maintain supersaturation levels for an extended period, and ultimately improve the bioavailability of poorly soluble drugs. Precipitation inhibitors prevalent in SDDSs include hydroxypropyl methylcellulose (HPMC), hydroxypropyl methylcellulose-acetate succinate (HPMCAS), polyvinyl pyrrolidone (PVP), and polyethylene glycol (PEG). These polymers inhibit drug precipitation by delaying crystalline growth or drug molecule nucleation [11]. Previous studies have demonstrated that supersaturation technology using PVP can overcome the pH-dependent solubility of poorly soluble drugs such as carbamazepine and indirubin [12,13]. Co-amorphizations based on molecular interactions between drugs and small molecular precipitation inhibitors have been explored for enhanced physical stability and dissolution. In these studies, flubendazole and indomethacin formed complexes with phenylalanine and tryptophan, respectively, and were maintained in stable supersaturated solutions [14,15]. While many studies have investigated the supersaturation phenomenon in the presence of a single precipitation inhibitor, studying drug precipitation under a combination of two precipitation inhibitors for poorly soluble drugs such as PZH has become necessary. Therefore, predicting and identifying precipitation inhibitor combinations is increasingly in demand [16].

In this study, a supersaturated liquid formulation (SSLF) loaded with PZH was developed to enhance solubility and dissolution rate. Liquid formulations have an advantage over solid formulations in terms of dissolution rate. The dissolution of solid dosage forms involves disintegration and erosion processes, whereas liquid formulations exhibit a faster dissolution rate with direct dispersion into the bulk medium compared with that of solid-dosage forms [17]. PVP was selected in a preliminary study as a precipitation inhibitor that was dissolved in glycerol, and its solubility was measured. The SSLF used comprised a simple liquid-state solution and a mixture of safe glycerol and precipitation inhibitors instead of solid dispersion and a SNEDDS. The optimized SSLF with a combination of precipitation inhibitors was investigated in terms of dispersion solubility and precipitation inhibition. Furthermore, the dissolution rates for SSLFs in media of varying pH were measured, and the ability to maintain supersaturation using different PZH supersaturation ratios was evaluated. Transmission electron microscopy (TEM) was performed to assess the performance of SSLFs in inhibiting PZH precipitation.

## 2. Results and Discussion

### 2.1. pH Solubility of PZH at 37 °C

The solubility of PZH in various buffer solutions at 37 °C, a normal body temperature, was determined and is summarized in Table 1. The average change in pH before and after adding raw PZH to the test medium was less than 0.10 in pH 1.2, pH 4.0, and pH 6.8 buffer solutions. However, in water, which lacks buffering capacity, the average pH decreased from 6.11 to 2.68 due to the release of protons from PZH dissociation. The solubility at pH 1.2 was the highest at 953.22 ± 34.16 μg/mL, approximately 170- and 435-fold higher than that at pH 4.0 (5.58 ± 0.28 μg/mL) and pH 6.8 (2.19 ± 0.08 μg/mL), respectively. In particular, the solubilities at pH 1.2 and pH 4.0 were higher at 37 °C compared with those in previously reported results at room temperature (682.64 ± 7.58 μg/mL at pH 1.2, 3.00 ± 0.25 μg/mL at pH 4.0), and the solubility ratio between pH 1.2 and 6.8 was larger at 37 °C than at room temperature (435-fold vs. 259-fold) [9]. PZH showed more pH-dependent solubility at 37 °C rather at room temperature. The solubility in water was 125.38 ± 28.51 μg/mL. The enhanced solubility observed at acidic pH 1.2 was attributed to the complete protonation and ionization of the indazole and pyrimidine structural groups, as their respective ionization constants (pKa = 2.1 and 6.4) exceed the pH of the buffer solution [18,19]. Conversely, PZH exhibited insolubility at pH 6.8, where non-ionized forms dominate due to the pH surpassing the pKa of 6.4 [9]. With increasing pH, solubility declined due to the decreased proportion of ionized forms. PZH demonstrated pronounced pH-dependent solubility (pH 1.2 >> water > pH 4.0 and pH 6.8) at 37 °C, necessitating strategies to maintain consistently high dissolution rates regardless of the medium’s pH [9].

**Figure 2:**
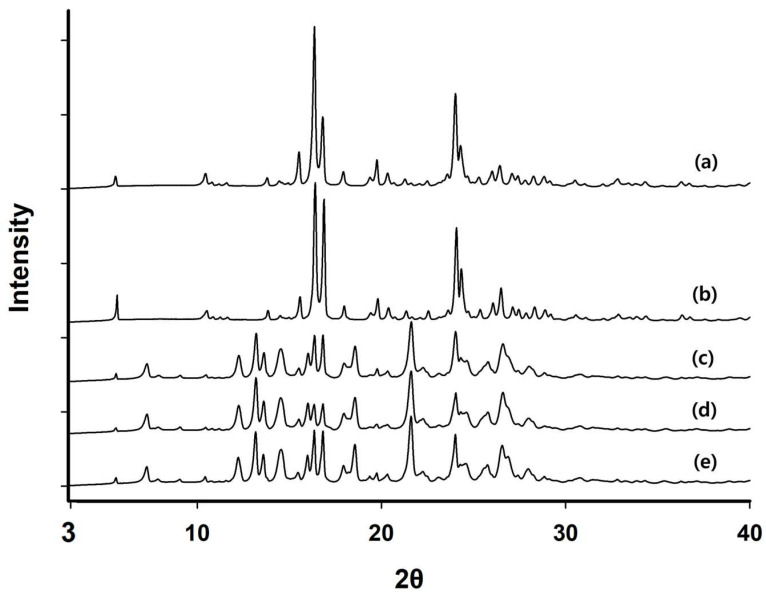
PXRD patterns of raw PZH (a), residual PZH obtained from 1.2 buffer (b), pH 4.0 buffer (c), pH 6.8 buffer (d), and water (e).

PXRD (powder X-ray diffraction) patterns of raw PZH and the residual PZH powders obtained from the pH solubility test are shown in Figure 2. The results indicated that the residual PZH in the pH 1.2 buffer solution showed the same PXRD pattern as the raw PZH powder. This suggests that the crystal form of PZH used in this work is stable at pH 1.2 and maintains its three-dimensional structural form. However, at pH levels of 4.0, 6.8, and in water, the residual PZHs showed identical PXRD patterns among themselves but different from that of the raw PZH powder, indicating a change in crystal form. As a result, PZH was highly dissolved at pH 1.2 while maintaining its hydrochloride salt form, but it changed to another crystal form at pH levels of 4.0, 6.8, and in water, resulting in poor solubility.

### 2.2. Solubility of PVP in Glycerol

**Figure 3:**
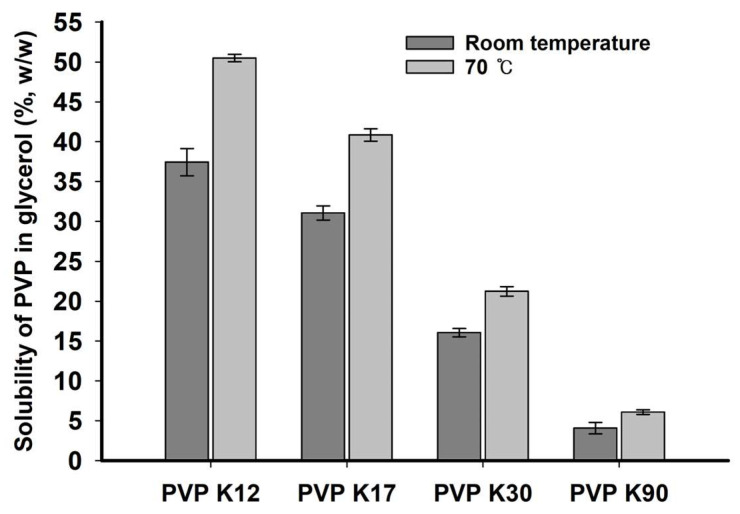
Solubility of PVPs in glycerol at room temperature and 70 °C.

The solubility of PVP in glycerol was determined at room temperature and 70 °C, and the results are shown in Figure 3. PVP solubility in glycerol was significantly higher at 70 °C than that at room temperature. The solubilities of PVP K12, K17, K30, and K90 were 50.49 ± 0.47%, 40.85 ± 0.78%, 21.25 ± 0.60%, and 6.08 ± 0.29% (*w*/*w*), respectively. The solubility of PVP in glycerol decreased proportionally with increasing PVP molecular weight. The higher intermolecular attraction and viscosity of high molecular weight PVP limited molecular dissolution and diffusion in bulk glycerol, resulting in lower glycerol solubility [20]. Therefore, heating to 70 °C was effective in decreasing glycerol viscosity and achieving high PVP solubility relative to that at room temperature. Molecular weights of not more than PVP K30 exhibited a solubility of >20% in glycerol. The solubility percentage of each PVP according to its molecular weight at 70 °C was considered for further studying PZH solubility in vehicles.

### 2.3. Solubility of PZH in Vehicles

**Figure 4:**
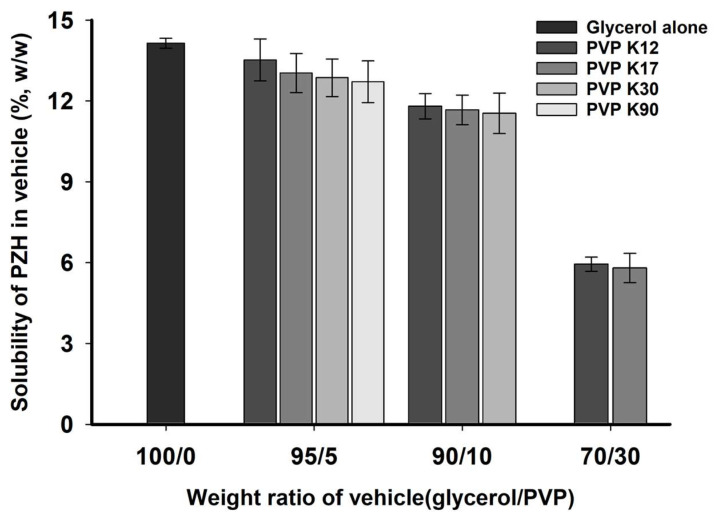
Solubility of PZH in vehicles at 70 °C.

The solubility of PZH in vehicles according to PVP molecular weight and its weight ratio was evaluated at 70 °C and is shown in Figure 4. The solubility of PZH in vehicles containing PVP was lower compared with that containing glycerol alone and decreased as the weight ratio of PVP increased from 5 to 30% (*w*/*w*) since glycerol is more effective in dissolving PZH than PVP. The increase in the viscosity of vehicles reduced the molecular dissolution and diffusion of PZH in the bulk vehicle on the surface of PZH drug particles, resulting in lower solubility compared with that in glycerol alone [21]. The PZH solubilities of PVP K12, K17, and K30 at a glycerol/PVP weight ratio = 90/10% (*w*/*w*) were 11.81 ± 0.47, 11.67 ± 0.55, and 11.54 ± 0.75% (*w*/*w*), respectively, showing solubilities of more than 10%. However, at a glycerol/PVP weight ratio = 70/30% (*w*/*w*), both PVP K12 and K17 exhibited low solubilities of less than 6% due to the higher PVP weight ratio and high viscosity. In the present study, the target loading percentage of PZH should be not less than 10% to reduce the vehicle weight ratio and total SSLF weight. Based on this criterion, the glycerol/PVP weight ratio = 70/30% (*w*/*w*) was excluded for further SSLF preparation.

### 2.4. Effect of PVP Molecular Weight on PZH Dispersion Solubility

**Figure 5:**
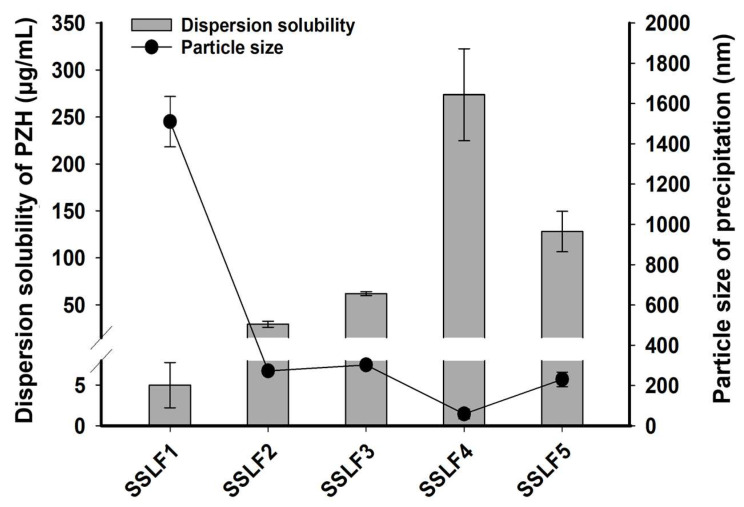
Dispersion solubility of PZH and particle size of PZH precipitates in SSLFs at pH 6.8.

To evaluate the precipitation inhibition of PZH in SSLF according to the molecular weight of PVP, the dispersion solubility of PZH and the particle size of its precipitate were measured, and the results are shown in Figure 5. The dispersion solubility of PZH for SSLF4 prepared with PVP K30 was the highest (273.66 ± 48.91 μg/mL) and was 54.73-fold higher than that of SSLF1 prepared with glycerol alone (5.00 ± 2.78 μg/mL). Moreover, the particle size of the PZH precipitate was smaller for SSLF4 (58.41 ± 23.79 nm) than that for SSLF1 (1510.37 ± 124.76 nm). The particle sizes of the PZH precipitates of SSLF2, SSLF3, and SSLF5 containing PVP K12, K17, and K90 were <500 nm and their dispersion solubilities were 29.31 ± 3.22, 61.85 ± 1.10, and 127.91 ± 21.51 μg/mL, respectively. PVP inhibited recrystallization initiation and growth by its positioning in buffer solution, adsorption on the PZH particles in solution, and formation of a physical barrier outside the particles, consequently increasing supersaturation solubility [11]. The ability of PVP to induce PZH supersaturation and inhibit crystal growth is attributed to the many hydrogen bond acceptors of PVP and its interaction with the hydrogen bond donor of PZH. PVP contains multiple hydrogen bond acceptors (e.g., carbonyl groups) within its polymer chains. Pazopanib includes hydrogen bond donors (e.g., amine groups), allowing for the formation of strong hydrogen bonds between PVP and pazopanib. These hydrogen bonds inhibit the recrystallization of pazopanib molecules and ensure their stable dispersion in the solution [19]. In addition, theoretical and experimental results generally corroborated the fact that higher molecular weight polymers more effectively maintained supersaturation than lower molecular weight polymers (PVP K30 > PVP K17 > PVP K12). The higher molecular weight of PVP increases the viscosity of the solution, reducing the mobility of the drug molecules. This contributes to maintaining pazopanib in a supersaturated state, as higher viscosity lowers the likelihood of the drug molecules meeting and forming crystals, thus prolonging the supersaturated condition [22]. However, for PVP K90 in SSLF5, a relatively low PVP weight ratio (PZH/glycerol/PVP K90 = 10/85/5, *w*/*w*) due to PVP’s low glycerol solubility limited the inhibition of PZH precipitation. As a result, PVP functioned as a PZH precipitation inhibitor, and PVP K30 in SSLF4 exhibited the most effective PZH precipitation inhibition and the highest PZH dispersion solubility.

### 2.5. Effect of PG on the Dispersion Solubility of PZH in the SSLF

**Figure 6:**
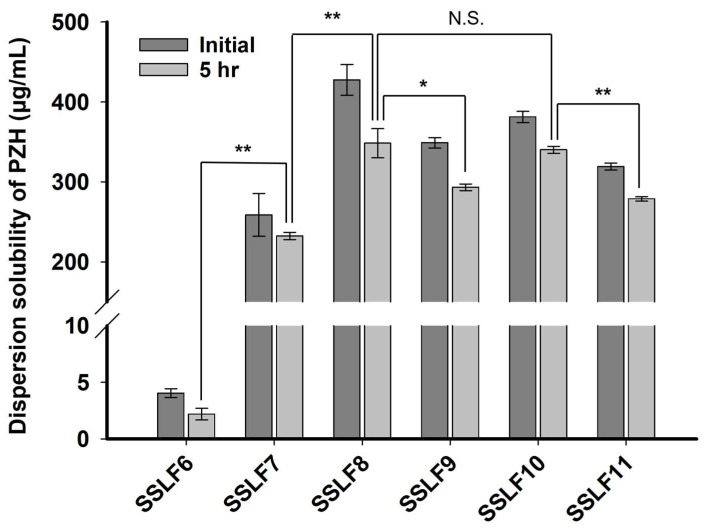
Dispersion solubility of PZH in SSLFs in pH 6.8 buffer solution at the initial time and 5 h. * *p* < 0.05; ** *p* < 0.005; N.S., no significant differences (*p* > 0.05).

PG (propylene glycol) is a general excipient used in pharmaceutical formulations due to its ability to improve drug solubility [23]. When mixed with glycerol, PG can interact differently with various drug molecules to improve their solubility. As shown in Figure 6, SSLF7 containing PVP K30 showed much higher PZH dispersion solubility (258.82 ± 26.80 and 232.49 ± 4.54 μg/mL initially and at 5 h, respectively) compared with that of SSLF6 without PVP K30 (4.03 ± 0.39 and 2.19 ± 0.51 μg/mL, respectively). The dispersion solubility of PZH for all SSLFs at 5 h was lower than that at the initial, owing to the gradual increase in PZH precipitation. Therefore, PZH dispersion solubility at 5 h was more important than that at the initial time since PZH precipitation was more static and closer to equilibrium at 5 h. In the comparison of SSLF7 and SSLF8, the combined use of PVP K30 and PG in SSLF8 (348.52 ± 18.19 μg/mL) significantly enhanced the dispersion solubility of PZH at 5 h compared with that in SSLF7 (232.49 ± 4.54 μg/mL) and the two ingredients in SSLF8 synergistically enhanced solubility and inhibited PZH precipitation. Such combinations can exhibit favorable effects on the precipitation inhibition of drugs, and previous research has demonstrated synergistic inhibition effects using PVP in combination with other types of precipitation inhibitors, flufenamic acid and nicotinamide, enhancing the supersaturation solubility of indomethacin [16,24]. In SSLF9, the higher PG weight ratio percentage suppressed PZH solubility compared with that in SSLF8, and the optimum PG weight ratio percentage was critical to establishing a synergistic effect between PVP K30 and PG. For SSLF10 and SSLF11, the higher loading of 10% PZH relative to that of SSLF6–9 at 5% PZH increased the precipitation rate. However, SSLF10 with an optimum weight ratio percentage of PVP K30 and PG showed no significant difference (*p* > 0.05) in the dispersion solubility of PZH at 5 h compared with SSLF8 (340.20 ± 4.35 μg/mL for SSLF10 and 348.52 ± 18.19 μg/mL for SSLF8). As a result, adding PG as a precipitation inhibitor worked synergistically with the PVP K30 and glycerol systems, and supersaturation stability was maintained even at a PZH ratio as high as 10%. Modification in the high viscosity and polarity of glycerol through the co-solvent system combining PG and glycerol ultimately would play a role to stabilize SSLF and to inhibit the PZH precipitate by reducing the crystallization speed of PZH along with PVP K30. In the case of SSLF10, PZH precipitation in the liquid formulation did not occur even after 3 months of storage at room temperature. Polymer-based precipitation inhibitors can increase the viscosity of liquid formulations, reducing the mobility of drug particles and consequently improving the physical stability of supersaturated formulations [25].

### 2.6. Dissolution Test

**Figure 7:**
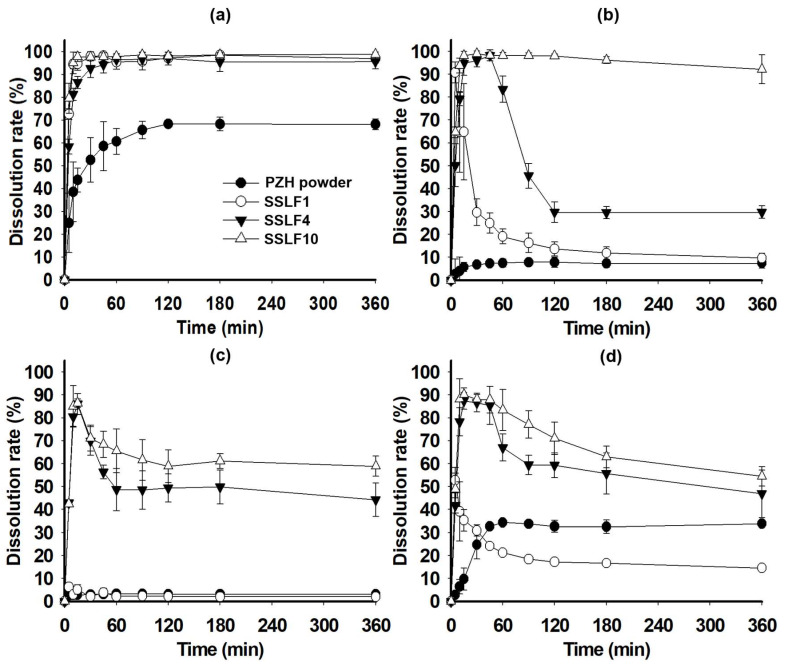
Dissolution profiles of SSLF1, SSLF4, SSLF10, and PZH powder in pH 1.2 buffer (**a**), pH 4.0 buffer (**b**), pH 6.8 buffer (**c**), and water (**d**).

The dissolution test was conducted for PZH powder, SSLF1, SSLF4, and SSLF10, as shown in Figure 7. The sink condition was only at pH 1.2 and the others (pH 4.0, pH 6.8, and water) were beyond the sink condition when the PZH concentration of 100% dissolution (111.11 μg/mL) was compared to each pH solubility of raw PZH as shown in Table 1. In pH 1.2 buffer solution, all SSLFs showed a complete dissolution rate of >90%, whereas PZH powder showed a low dissolution rate (68.19 ± 2.31%) at 360 min (Figure 7a). In pH 4.0 buffer solution, SSLF10 maintained a high and complete dissolution rate of >90% until 360 min. However, SSLF1 and SSLF4 exhibited a spring effect and low dissolution rate of <40% at 360 min (Figure 7b), which was due to relatively rapid crystallization and precipitation of PZH upon exposure to the external buffer solution [26]. In pH 6.8 buffer solution, the dissolution rates at 360 min were in the order of SSLF10 (58.90 ± 4.41%) > SSLF4 (44.28 ± 7.32%) >> SSLF1(2.05 ± 0.11%) and PZH powder (3.17 ± 0.10%) (Figure 7c). The dissolution rates for PZH powder and SSLF1 were very poor in pH 6.8 buffer solution at 37 °C owing to inferior pH solubility and rapid PZH precipitation. However, SSLF4 and SSLF10 containing the PZH precipitation inhibitors, PVP K30 with or without PG, exhibited a parachute effect and high dissolution rate. In water, SSLF4 and SSLF10 showed higher dissolution rates than those at pH 6.8 (Figure 7d). From these results, SSLF4 and SSLF10 were shown to induce supersaturation immediately after dispersion in the buffer solution and simultaneously inhibited crystal growth and precipitation under the influence of precipitation inhibitors such as PVP K30 and PG. Notably, according to previous studies, the commercial product (Votrient^®^) exhibited very low dissolution rates (77.91 ± 3.95% at pH 1.2, 48.72 ± 4.93% at pH 4.0, 15.24 ± 0.38% at pH 6.8) until 120 min [3,8]. SSLF10 exhibited superior dissolution rates in all pH ranges compared with those of the commercial product, which could be attributed to their enhanced bioavailability relative to that of the commercial product.

### 2.7. Supersaturation Ratio

**Figure 8:**
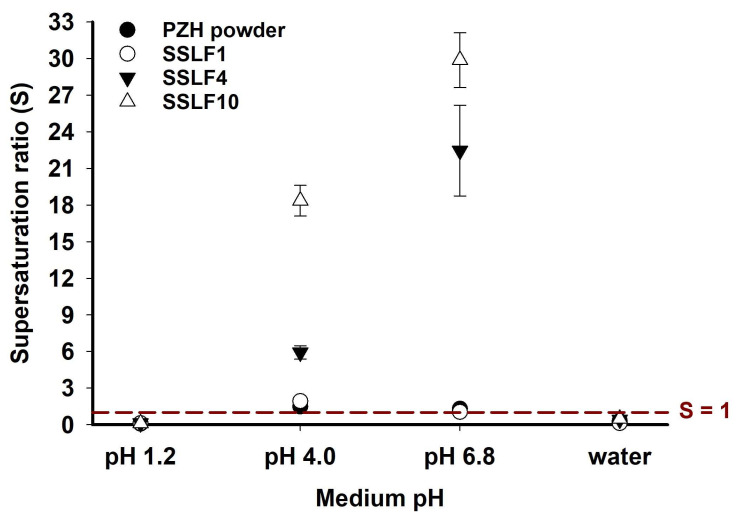
Supersaturation ratio (S) of SSLF1, SSLF4, SSLF10, and PZH powder from the dissolution rate at 360 min in pH 6.8 buffer solution as in Figure 7.

The supersaturation ratio (S) indicates the extent to which PZH supersaturates in SSLFs in various dissolution test mediums [27]. The calculated S values are shown in Figure 8. A higher S implies that the PZH solution was in a more supersaturated state compared with that of a simple pH solubility solution. The theoretical maximum PZH concentration in the dissolution test was 111.11 μg/mL (=100 mg PZH/900 mL) when 100% of the PZH in all SSLFs and PZH powder was dissolved; the theoretical maximum supersaturation ratios (S) were 0.12, 19.91, 50.73, and 0.89 at pH 1.2, 4.0, and 6.8, and water, respectively. Despite the poor pH solubility of PZH at pH 4.0 and 6.8, SSLF10 and SSLF4 showed high S values of >5. Moreover, SSLF10 exhibited the highest S values of 18.36 and 29.88 at pH 4.0 and 6.8, respectively. PVP K30 and PG in SSLF10 synergistically enhanced supersaturation solubility and inhibited PZH precipitation. On the other hand, both SSLF1 and PZH powder at pH 4.0 and 6.8 had similar S values close to 1 due to poor pH solubility and dissolution rate, indicating that glycerol alone did not increase the S value [28]. At pH 1.2 and 4.0, the S values for all SSLFs and PZH powder were much lower than 1 since the pH solubility of PZH at 37 °C was much higher than the theoretical maximum dissolution concentration of PZH. As a result, SSLF10 exhibited a stable PZH supersaturation state and enhanced the dissolution rate with the inhibition of PZH precipitation and recrystallization. From these results, utilizing PVP K30 and liquid PG as a hydrophilic polymer and solvent, respectively, exhibited a synergistic effect in inhibiting PZH precipitation without compromising solubility. The synergistic mechanism by which PG addition to PVP K30 enhances pazopanib’s precipitation inhibition involves several factors. Firstly, there the co-solvent effect of PG, which, when mixed with water, increases pazopanib’s solubility due to its hydrophilic and lipophilic properties, interacting with pazopanib’s nonpolar regions [29]. Secondly, PG enhances hydrogen bonding by acting as both a hydrogen bond donor and acceptor, forming additional bonds with pazopanib and PVP K30, thus inhibiting recrystallization and maintaining a supersaturated state [19]. Lastly, the mixed solvent system of PVP K30 and PG provides a synergistic effect. PVP K30 forms a physical barrier, while PG enhances solubility, collectively improving pazopanib’s solubility and stability, allowing the drug to remain stable in solution [30]. These mechanisms help maintain the supersaturated state of pazopanib, maximizing the precipitation inhibition effect, and thus enhancing its solubility and bioavailability. The formulation design approach employing this combination can be applied to other poorly soluble drugs.

### 2.8. TEM Analysis

**Figure 9:**
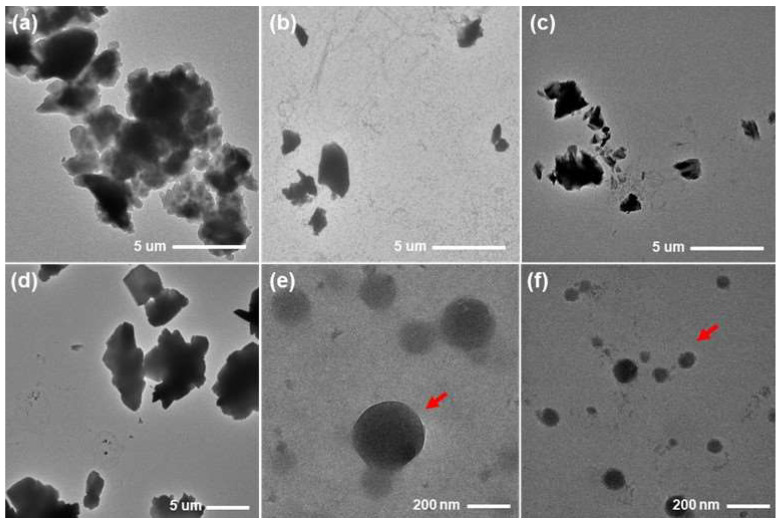
Transmission electron microscopy (TEM) images of PZH powder (**a**) and PZH powder dispersed in water (**b**) and pH 6.8 buffer solution (**c**); precipitation particles from the dissolution test of SSLF1 (**d**), SSLF4 (**e**), and SSLF10 (**f**) at 2 h in pH 6.8 buffer solution.

In the TEM image analysis, PZH powder showed short rod-shaped crystalline characteristics and particle sizes larger than several μm with an opaque internal state (Figure 9a). The dispersed particles of PZH powder in water and pH 6.8 buffer solution were similar to those of the original PZH powder, and no critical change in the shape and particle size due to the exposure of PZH powder to the aqueous medium was observed (Figure 9b,c). SSLF1 resulted in large crystallized particles with very similar morphology to the original PZH powder, strongly supporting the conclusion that the particles were from the recrystallization of PZH molecules solubilized in glycerol when exposed to the pH 6.8 buffer solution, an anti-solvent. Thus, glycerol alone did not inhibit PZH recrystallization and precipitation. In the particles from SSLF4 and SSLF10, spherical particles (red arrows) of <300 nm were observed (Figure 9e,f), and the typical crystalline morphology as that in the original PZH powder and SSLF1 was absent. Moreover, SSLF10 showed smaller spherical particles of <200 nm compared with those of SSLF4, with the synergistic effect of PVP K30 and PG on PZH recrystallization inhibition. It was presumed that the SSLF10 precipitation particles exhibited an amorphous state of PZH rather than the crystalline form, based on the observed differences in morphology. These TEM analysis observations suggest that PVP K30 and PG in SSLF10 are very effective PZH precipitation inhibitors and dissolve a large amount of PZH in the supersaturation state, which may explain the high dissolution rates regardless of dissolution medium pH [27].

## 3. Materials and Methods

### 3.1. Materials

PZH was purchased from Hangzhou Royall Import and Export Co., Ltd. (Hangzhou, China). The PZH used in this work was in anhydrous crystalline form with a beginning melting point of approximately 300 °C. Propylene glycol (PG) and polyvinylpyrrolidone K12 (PVP K12), K17 (PVP K17), K30 (PVP K30), and K90 (PVP K90) were kindly provided by BASF (Ludwigshafen, Germany). Glycerol was purchased from Duksan Pure Chemicals (Ansan, Republic of Korea). All other chemicals were of reagent grade and used without further purification.

### 3.2. HPLC Conditions

The determination of PZH concentrations in the sample solutions was carried out through the utilization of high-performance liquid chromatography (HPLC) according to the previous method by Choi et al. Briefly, the analysis of PZH in the samples was executed employing a Waters 2695 HPLC system (Waters Corporation, Milford, MA, USA) equipped with a UV–Vis detector (Waters 2487; Waters Corporation). Separation of PZH was achieved using a reverse phase column (C18 column, 5 μm, 4.6 × 150 mm; Osaka Soda, Osaka, Japan). The mobile phase consisted of a mixture of 0.02 M ammonium acetate aqueous solution (pH 7.0), acetonitrile, and methanol (47/37/16, *v*/*v*/*v*). HPLC analysis was conducted at a flow rate of 1.0 mL/min, with an injected volume of 10 μL, and UV detection at 260 nm. The acquired data were processed using Waters LC Solutions software (version 2.0; Waters Corporation).

### 3.3. pH Solubility of PZH at 37 °C and Powder X-Ray Diffraction (PXRD) Characterization

The pH solubility of PZH at 37 °C was evaluated in HCl/NaCl (pH 1.2), acetate (pH 4.0), and potassium phosphate buffer solution (pH 6.8) and distilled water. An excess of PZH (approximately 15 mg) was added to 10 mL of the prepared buffer solutions and water in a vial and stirred at 500 rpm in a water bath at 37 °C for 24 h. Next, each mixture was aliquoted at 1 mL, transferred to a tube, and centrifuged (LZ-1730R; LABOGENE, Seoul, Korea) at 15,000 rpm for 30 min. Thereafter, 0.5 mL of the supernatant was diluted 5-fold with methanol and filtered through a syringe filter (0.45-μm pore size, DISMIC^®^-13HP; ADVANTEC, Tokyo, Japan). The concentration of PZH in the filtrate was determined using the aforementioned HPLC conditions.

To check if there was any crystal form change, residual PZH powder was obtained from the intended same treatment as the above pH solubility test with scale up to an amount of 300 mg of PZH. PXRD characterization of the sample (raw PZH, residual PZH powders obtained from pH 1.2, pH 4.0, pH 6.8 buffers and water) was recorded using an X-ray diffractometer (Rigaku IV Ultima; Tokyo, Japan), which was equipped with a Linxeye 1-D detector. Each sample was added to the grid, and the diffraction pattern of each sample was measured using a Cu Kα radiation source (40 kV and 40 mA) with an acquisition time of 0.2 s per step. The scanning range was 5°~40° in the 2θ range.

### 3.4. Solubility of PVP in Glycerol

The solubility of PVP in glycerol was measured at room temperature and 70 °C. The solubility of PVP at room temperature was determined by placing PVP (500 mg) in a vial containing 500 mg of glycerol, stirring at 300 rpm for 6 h at room temperature, and observing whether PVP completely dissolved. If PVP remained, an exactly weighed 30 mg of glycerol was added and stirred for 5 min. This procedure was repeated until PVP was dissolved completely. The solubility of PVP (%, *w*/*w*) in glycerol was calculated from the total amounts (mg) of glycerol and PVP. The criterion for the complete dissolution of PVP was a clear liquid state with no insoluble particles or lumps when visually observed under light. The solubility of PVP at 70 °C was determined in a heated silicon oil bath by adding PVP (600 mg) to a vial containing 500 mg of glycerol and stirring at 300 rpm for 6 h. The remaining procedures were the same as those for room temperature conditions.

### 3.5. Solubility of PZH in the PVP and Glycerol (Vehicle) Solution

The solubility of PZH in vehicles of glycerol and various molecular weight PVPs (PVP K12, PVP K17, PVP K30, and PVP K90) was determined. Each vehicle solution according to the weight ratio of glycerol/PVP (=100/0, 95/5, 90/10, or 70/30; *w*/*w*) was prepared by stirring the mixture of PVP and glycerol to transparency at 70 °C in a heated silicon oil bath for 30 min. An excess of PZH powder (approximately 300 mg) was weighed and added to a vial containing 1 g of the prepared vehicle and stirred at 70 °C for 5 h. Subsequently, approximately 1 g of each mixture was transferred to a tube and centrifuged (LZ-1730R; LABOGENE) at 15,000 rpm for 30 min. Thereafter, 0.5 mL of the supernatant was diluted 5-fold with methanol and filtered through a syringe filter (0.45-μm pore size, DISMIC^®^-13HP; ADVANTEC). The concentration of PZH in the filtrate was determined using the aforementioned HPLC conditions.

### 3.6. Preparation of Supersaturated Liquid Formulations

SSLFs were prepared at the compositions shown in Table 2. SSLF1 was prepared by stirring PZH in glycerol alone at 70 °C for 30 min until PZH was completely dissolved. All the other SSLFs, i.e., SSLF2–11, were prepared by stirring the mixture of PVP and glycerol in a vial at 70 °C for 30 min. PG was added and stirred in for 30 min at 70 °C only for SSLF8–11. Next, PZH was added to the prepared vehicle and stirred at 70 °C for 30 min until PZH was completely dissolved. We confirmed, using an HPLC chromatogram, that PZH remains stable without chemical degradation when solubilized and processed at 70 °C. The total preparation scale of each SSLF batch was set to 10 g, and the amount of each ingredient according to the composition percentage in Table 2 was calculated and used.

### 3.7. Dispersion Solubility of PZH

The dispersion solubility of PZH for SSLF was measured in the pH 6.8 phosphate buffer solution in which PZH dissolved poorly. One gram of the prepared SSLF (equivalent to 50 or 100 mg PZH) was added to 5 mL of pH 6.8 phosphate buffer solution in a vial with vortexing for 1 min. This solution was transferred to a tube and centrifuged (LZ-1730R; LABOGENE) at 15,000 rpm for 30 min, and then 0.5 mL of the supernatant was diluted 5-fold with methanol and filtered through a syringe filter (0.45-μm pore size, DISMIC^®^-13HP; ADVANTEC). The concentration of PZH in the filtrate was determined using the aforementioned HPLC conditions. The dispersion solubility of PZH referred to the concentration of PZH at room temperature measured by rapidly dispersing SSLF in a pH 6.8 phosphate buffer solution with a vortexing for 1 min.

### 3.8. Particle Size of Precipitates

The determination of particle size for PZH precipitates resulting from the evaluation of PZH dispersion solubility in SSLF1–5 was conducted using a particle size analyzer (NanoBrook 90Plus; Brookhaven Instruments Corporation, Holtsville, NY, USA). One milliliter of the samples from the PZH dispersion solubility tests was diluted by a 49 mL of pH 6.8 phosphate buffer solution in a vial with vortexing for 30 s. Measurements were taken at a wavelength of 659 nm and a scattering angle of 90°, with an analysis temperature set to 25 °C and the number of measurements performed over 5 cycles. The process of particle size measurement was repeated three times for accuracy.

### 3.9. Dissolution Studies

The dissolution test was performed using a 708-DS dissolution tester (Agilent Technologies, Santa Clara, CA, USA), and the dissolution media were 900 mL of pH 1.2, 4.0, and 6.8 buffers and water. The dissolution test conditions comprised the paddle method at 50 rpm and 37 ± 0.5 °C. SSLFs filled in #0 hard gelatin capsules corresponding to 100 mg of PZH were placed in the dissolution tester with a sinker. Afterward, samples were aliquoted at 5 mL using a syringe at predetermined time intervals (5, 10, 15, 30, 45, 60, 90, 120, 180, and 360 min). Next, the aliquots were diluted 4-fold with diluent (methanol) and filtered through a syringe filter (0.45-μm pore size, DISMIC^®^-13HP; ADVANTEC). The concentration of PZH in the filtrate was determined using the aforementioned HPLC conditions.

### 3.10. Drug Supersaturation Ratio (S)

The ability of SSLF to remain supersaturated in solution without PZH precipitation during the dissolution test was evaluated using the supersaturation ratio (S). The supersaturation ratio was calculated using Equation (1). The equation is as follows:

S = C_Dissolution rate at 360 min_/C_PZH_, (1)

where C_Dissolution rate at 360 min_ is the concentration of PZH (μg/mL) calculated from each final dissolution rate at 360 min in Figure 7, and C_PZH_ is the saturated pH solubility (μg/mL) of PZH at 37 °C obtained from Table 1. The dissolution rate measurement time for calculating the supersaturation ratio was selected and compared at the final time point of the dissolution test, 360 min, when the difference between compositions can be best observed.

### 3.11. Transmission Electron Microscopy (TEM) Analysis

The particle size and shape of PZH powder, PZH powder dispersed in water and pH 6.8 buffer solution, and precipitate particles obtained from SSLF1, SSLF4, and SSLF10 at 2 h of the dissolution test in pH 6.8 buffer solution were analyzed using a transmission electron microscope (Talos L120C; Thermo Fisher Scientific, Waltham, MA, USA). Samples of the liquid state were obtained and dropped onto a carbon-coated TEM grid (TF300C; TMA Co., Ltd., Seoul, Korea) and dried for 24 h at room temperature. All measurements were performed at a voltage of 120 kV and room temperature.

### 3.12. Statistical Analysis

Experiments were performed in triplicate, and data were expressed as mean ± standard deviation. Student’s *t*-test or one-way ANOVA was used to determine statistically significant differences among groups. Statistical analyses were performed using Prism software (ver. 10.0; GraphPad Software, La Jolla, CA, USA). *p* < 0.05 or *p* < 0.005 was considered statistically significant.

## 4. Conclusions

PZH exhibited highly pH-dependent solubility at 37 °C and insolubility in pH 4.0 and 6.8 buffer solutions. An SSLF was developed to increase PZH solubility and dissolution rate. The SSLF was prepared at 70 °C with glycerol as a solvent and PVP K30 and/or PG as precipitation inhibitors. In the characterization including dispersion solubility and particle size of PZH precipitates, the weight ratio percentage of PVP K30 and PG was optimized in SSLF10 (PZH/glycerol/PG/PVP K30 = 10/50/20/20, *w*/*w*) with a high PZH loading of ≥10%, precipitated particle size of <300 nm, and consistent high dissolution rate at pH 4.0 (>90%) and 6.8 (>55%) until 360 min. The supersaturation ratios of SSLF10 were 18.36 and 29.88 at pH 4.0 and 6.8, respectively. The TEM imaging of SSLF10 showed clear evidence for the inhibition of PZH precipitation with amorphous small PZH particles. In this work, SSLF10 is considered a promising formulation candidate with enhanced solubility and dissolution rate regardless of pH and has potential implications for formulating supersaturated solutions of other low-solubility drugs. However, long-term stability tests and comparisons to other solubilizing technologies will be needed in the future.

## Figures and Tables

**Table 1:** pH solubility of PZH at 37 °C.

Test Solution	Solubility at 37 °C (μg/mL)
pH 1.2 buffer	953.22 ± 34.16
pH 4.0 buffer	5.58 ± 0.28
pH 6.8 buffer	2.19 ± 0.08
water	125.38 ± 28.51

Data are presented as the means ± standard deviation (*n* = 3).

**Table 2:** The composition ratio percentages of SSLFs.

SSLF	Composition Ratio (%, *w*/*w*)						
	PZH	Glycerol	PG	PVP K12	PVP K17	PVP K30	PVP K90
SSLF1	10	90	0	0	0	0	0
SSLF2	10	70	0	20	0	0	0
SSLF3	10	70	0	0	20	0	0
SSLF4	10	70	0	0	0	20	0
SSLF5	10	85	0	0	0	0	5
SSLF6	5	95	0	0	0	0	0
SSLF7	5	75	0	0	0	20	0
SSLF8	5	55	20	0	0	20	0
SSLF9	5	40	35	0	0	20	0
SSLF10	10	50	20	0	0	20	0
SSLF11	10	35	35	0	0	20	0

## Data Availability

Data are contained within the article.
